# Effect of Montelukast 10 mg in Elderly Patients with Mild and Moderate Asthma Compared with Young Adults. Results of a Cohort Study

**DOI:** 10.2174/1874306401812010067

**Published:** 2018-11-14

**Authors:** Guillermo Sánchez, Diana Buitrago

**Affiliations:** 1 Fundación Universitaria de Ciencias de la salud, SIIES: Research and Education in Health, Bogotá, Colombia; 2 Department of Research, SIIES: Research and Education in Health, Bogotá, Colombia

**Keywords:** Asthma, Leukotriene antagonists, Anti-asthmatics, Montelukast, Elderly, Young adult

## Abstract

**Background:**

The clinical characteristics and physio-pathogenic mechanisms of asthma in patients older than 60 years appear to differ from the behavior described for other age groups. Therefore, the effectiveness of medications for elderly patients with asthma should not be extrapolated from studies conducted on teenagers or young adults.

**Objective:**

The study aimed **** to establish the clinical effect of montelukast 10 mg in elderly patients with mild and moderate asthma compared to its effect on young adults.

**Method:**

A prospective cohort study was conducted during 12 weeks of follow-up, which consecutively included the total population of adult patients attended by a group of 21 general practitioners, between July and December 2016. Young adults (18-59 years) and older adults were included (60 years or older) with mild or moderate asthma, which, according to the criteria of his treating physician, had been prescribed montelukast 10 mg/day. The variables of interest were: use of inhaled corticosteroids during the last month, use of inhaled beta-2 adrenergic agonists as a rescue in the last month, having attended the emergency service during the last month due to an asthma attack, presence of wheezing in the physical examination, the number of attacks in the last month and the number of days without symptoms in the last month.

**Results:**

A total of 126 patients entered the cohort and 104 completed the follow-up, of which 29% were older adults. On admission, 65.4% of patients (68/104) had used rescue inhaled beta2 in the last month and had been using schemes with corticosteroids. After 12 weeks of follow-up, 58.1% (43/74) of the young adults required treatment schedules with corticosteroids, while in the elderly, only 36.7% of the patients (11/30) required this treatment scheme (*p*-value: 0.047). Regarding the use of rescue inhaled beta-2 at 12 weeks, 55% of young adults reported using them, compared to 33.3% of older adults (*p*-value: 0.041).

**Conclusion:**

In this cohort of patients, treated with montelukast 10 mg/day for 12 weeks, there was a reduction of broncho-obstructive symptoms and exacerbations of the disease. In older adults compared to young adults, a greater reduction in the use of beta2 agonists rescue medications and in the concomitant use of inhaled corticosteroid schemes was documented.

## 1 INTRODUCTION

The prevalence of asthma has been duplicating in the world every 10 years, and at present, it is estimated that about 300 million people suffer from this disease [1]. The country with the highest prevalence reported is Australia, with 32.8%, and in Latin America, Brazil stands out with reports close to 13% of cases diagnosed, and 24% of prevalence of symptoms compatible with asthma [2]. Specifically, in Colombia, it has been documented that the prevalence of symptoms is around 12% and the percentage of cases diagnosed is 7% [3]. This condition is not only responsible for a significant burden in terms of morbidity but also causes around 180,000 deaths per year in the world, among which a high percentage is considered preventable, mainly in the population of adults over 45 years [1]. It is worth noting that in Colombia about 35% of patients with asthma are older than 60 years, and in this population, the sub-diagnosis can be up to 69% [4].

It is known that, in the adult population, asthma is a growing problem, which can probably have an important underestimation phenomenon in these age groups, partly due to the confusion that can arise in the diagnosis of Chronic Obstructive Pulmonary Disease (COPD), or because of the coexistence of the two entities in the so-called “overlap syndrome” [5, 6]. The importance of the situation in the group of older adults with asthma is that, generally, in addition to the sub-estimation of their frequency, the mortality rate is higher than that of the population in the same age group, but who do not suffer from asthma [7]. In fact, it has been proposed that the pathophysiology of the disease in adults is different from that presented in children, which poses greater challenges for its treatment and adequate control [8]. It has been described that, in older adults, pharmacological treatments have different responses, including resistance to corticosteroids and better response to leukotriene receptor antagonists [7]. This behavior could be explained by the fact that, in these patients, there is a marked component of bronchoconstriction, edema of the airway and mucus formation, mediated by the role of leukotrienes, with lower levels of Ig-E and less frequency of concomitant cases of allergic rhinitis or atopic dermatitis [7, 9]. It is for this reason that it is important to document the effects of asthma treatments, not only in children, adolescents or young adults, but it is also important to know its effects on the elderly population, which has usually been excluded from controlled clinical trials [10].

There are multiple treatment alternatives for the different types and degrees of severity of asthma, including leukotriene receptor antagonists, among which is montelukast, which acts by blocking the action of leukotriene D4 on the leukotriene cysteine receptor - CysLT1 in the lungs and bronchi, causing a reduction in the broncho-obstructive and inflammatory component [11]. Different studies have shown how montelukast is able to reduce the symptoms that accompany asthma and its exacerbations, and to reduce the need to use rescue bronchodilators [11, 12]. However, as noted previously, in different clinical trials, the population of older adults has been excluded and the management protocols of this population group have generally been extrapolated from the results obtained in adolescents or young adults. Therefore, it is essential to have an evidence that, in real life conditions, can account for the true effectiveness of treatments in the adult population, including patients over 60 years of age.

Consequently, and based on the above approaches, the present investigation was developed with the objective of establishing the clinical effect of montelukast 10 mg/day in elderly patients with mild and moderate asthma compared to its effect in young adults.

## 2 MATERIAL AND METHODS

### 2.1 Design, Population and Sample

An analytical prospective cohort study was carried out, including consecutively the total population of adult patients attended by a group of 21 general practitioners, between July and December 2016, in 12 cities of Colombia, with diagnostic of asthma confirmed by clinical findings and spirometry, which at the time of the consultation were classified as mild or moderate asthma, according to the criteria proposed by GlNA 2017 (Global Initiative for Asthma-2017) [13], and who were candidates to be treated -according to medical criteria- with montelukast 10 mg day [14].

### 2.2 Procedure for Enrollment and Follow-Up

In real-life conditions, each doctor gave attention to their patient, made the corresponding diagnosis and assigned the treatment according to their clinical criteria. When the patient was considered a candidate to be part of the study, the doctor requested their informed consent to be able to document and analyze the clinical evolution data of their condition, during a period of 12 weeks. In each case, the professional prescribed the treatment according to their clinical criteria and allowed the research group to know the baseline and follow-up data. Clinical control appointments were defined by the attending physician, but for the purposes of the analysis, the last available control was recorded at week 12 of follow-up (± 2 weeks).

### 2.3 Variables of Interest

Sociodemographic variables were evaluated. A reclassification of the age variable was established as an independent variable, considering as young adults people between 18 and 59 years old, and older adults, patients with 60 years or older. Dependent variables were the use of inhaled corticosteroids as part of the routine treatment scheme during the last month, the need to use inhaled beta-2 adrenergic agonist as a rescue in the last month, having attended the emergency service during the last month during an asthmatic attack, the presence of wheezing in the physical examination, the number of attacks in the last month and the number of days free of symptoms in the last month. Using the change in clinical symptoms, we built the main outcome: overall improvement, defined as a patient who at the end of the follow - up had a total improvement in symptoms. Additionally, serious adverse events, defined as hospitalizations, mortality and serious infections, were described.

### 2.4 Statistical Analysis

A general description of the study variables was made, using measures of frequency, central tendency measures and dispersion statistics according to the scale of measurement. A baseline evaluation of the dependent variables was performed and compared with the results obtained after 12 weeks of follow-up. The results achieved at the end of follow-up were compared between young adults and older adults. In the dependent variables, on a qualitative measurement scale, proportions were compared at the end of follow-up between the two age groups. For the comparison of the dependent variables in numerical scale, the non-parametric Wilcoxon rank-sum test was applied. With categorical variables, the Chi-square test was performed. Cumulated incidence and risk ratio with a confidence interval of 95%, were calculated for the overall improvement, comparing young adults and older adults. Serious adverse events were described through absolute and relative frequencies. For hypothesis testing, an alpha value of 0.05 was defined, considering that a *p*-value below this cut-off point would be statistically significant.

## 3 RESULTS

A total of 126 patients diagnosed with mild to moderate asthma were included in the registry, of whom 104 patients attended the medical follow-up at week 12 (± 2 weeks), for a percentage of adherence with the medical check-up of 82.5%. Thirty-six percent were men (37/104) and the ages of the patients ranged between 18 and 96 years, with a median of 36.5 years and 50% of the subjects between 24.5 and 65 years. For purposes of comparison, the cohort was classified into two age groups: young adults 71% (74/104), and older adults 29% (30/104). The comparison of baseline parameters for young adult and elderly patients is presented in Table **1**.

### 3.1 Clinical Performance

During the baseline assessment, it was documented that 68 of the 104 subjects used steroid schemes and had used rescue inhaled beta-2 adrenergic agonist in the last month (65.4%). Additionally, in 13.5% (14/104), a history of emergency room visits was established in the last month, and in 49% of the cases (51/104), wheezing was detected by physical examination. The median of attacks in the last month was 2 (IQR: 1) and that of symptom-free days in the last month of 15 (IQR: 6.5).

After 12 weeks of follow-up, 51.9% of patients (54/104) required concomitant treatment with corticosteroids, while 49% had used rescue inhaled beta-2 adrenergic agonist (51/104). On physical examination, wheezing was documented in 30 of the 104 patients (28.9%) and 8.7% went to the emergency room due to asthma exacerbation (9/104). Regarding the median of seizures in the last month, it reached 0 (IQR: 0), and the median of symptom-free days in the last month rose to 24.5 (IQR: 10). Table **2** shows the findings of the entire cohort, comparing the baseline situation with the findings of the twelfth week. Differences were documented between baseline and 12 weeks of follow-up, in these variables: treatment with corticosteroids in the last month, use of rescue inhaled B2 in the last month, wheezing in the consultation, attacks in the past month, and symptom-free days in the last month (*p*-value <0,05).

We compared the clinical effect achieved after 12 weeks of treatment, in older adults and young adults. Differences were established in the use of rescue inhaled beta-2 adrenergic agonist in the last month and in the use of corticosteroids in the treatment scheme (*p* value <0.05). The results are presented in Table **3**.

The cumulative incidence of the overall improvement of the entire cohort was 47% (IC 95%: 37-57), in the arm of older adults was 63%, and in the young adults was 40.5% (RR: 1.53; IC 95%: 1.1-2.3; p value:0.03).

### 3.2 Serious Adverse Events

During the follow-up, no serious adverse events were recorded in any of the included patients and in no case was the suspension of treatment documented due to an adverse event.

## 4 DISCUSSION

Many of the treatments for the management of asthma in older adults have been extrapolated from the evaluation of effectiveness performed in patients in other age ranges, since the volume of evidence that has studied the effect of different therapeutic alternatives in patients in this age group is scarce, even for medications such as inhaled corticosteroids, which are recommended as the anti-inflammatory agents of the first choice in patients with asthma or for other alternatives such as leukotriene receptor antagonists [10, 14-16].

This is how the present investigation provides relevant evidence, from a study of drug use in real life conditions, in which a group of general practitioners prescribed montelukast 10 mg/day to patients in different age ranges, including older adults. The results obtained are relevant to the extent that, in the first place, it was possible to establish a significant clinical effect, in a series of parameters that were routinely evaluated in the consultation of this group of physicians, and secondly, the clinical effect was compared reached in young adults and older adults.

Based on the results obtained, it was possible to document a reduction in the percentage of patients who required concomitant management with inhaled corticosteroids, while reducing broncho-obstructive symptoms and exacerbations in all age groups. These findings are consistent with the properties attributed to leukotriene receptor antagonist drugs, and contribute with evidence that supports their prescription in cases of patients with mild to moderate asthma, either as an alternative to monotherapy, or in combination with other therapies such as inhaled corticosteroids, but seeking to reduce the required dose of the latter [7]. It is worth noting that, according to the results obtained, a percentage close to 13.5% of our patients managed to reduce the concomitant use of therapy with inhaled corticosteroids.

As previously mentioned, the main objective of the present investigation was to compare the clinical effect of montelukast 10 mg day in elderly and young adult patients. The results obtained, after 12 weeks of treatment, showed a positive clinical effect in the two groups, but with a greater reduction in the use of beta-2 adrenergic agonist as a rescue drug and in the concomitant use of schemes with inhaled corticosteroids in elderly adults. The overall improvement was significant for the entire cohort, but more importantly, the incidence of overall improvement in the older adults was 1.53 times the overall improvement in young adults. These results should be weighted considering that it has been documented that patients in this age group are more tolerant of their symptoms, and usually report a superior result when compared with young adults [17]. Notwithstanding the above, it is worth noting that the parameters that were positively modified in the elderly had not shown differences in the baseline measurement between young adult and elderly patients, so we concluded that they could be attributed to the effect of montelukast. In fact, the results obtained in the present investigation are consistent with the findings of Bozek *et al*., who compared the efficacy of montelukast in patients older than 60 years, with severe asthma symptoms, in treatment with inhaled corticosteroid therapy and could establish an improvement in the control of the clinical symptoms of asthma, and a reduction in the days of use of therapy with beta-2 adrenergic agonist [16].

In addition, the effectiveness of montelukast has also been evaluated under conditions similar to real life, in studies such as Virchow *et al*., which included 5855 individuals with asthma, in age ranges between 16 and 96 years, finding significant clinical results regarding the improvement of asthma symptoms, as has been documented in the present investigation, but with the particularity, that the results were not compared by age group [18].

At present it is known that there are a number of factors that must be taken into account when prescribing therapies in elderly people, since it is usually patients with other chronic diseases and who may also suffer from visual alterations, coordination, or disabilities of another nature that could prevent an adequate use of inhaled medications, so that oral medications, taken once a day, could influence greater control of symptoms, due to the simple effect of therapeutic adherence [19, 20]. Consequently, it is important to consider this aspect in the treatment of older adults with asthma, since the clinical effectiveness of a drug such as montelukast could be enhanced, by the ease of its posology once a day and without the difficulty which may represent the use of inhalers, especially in elderly patients, guaranteeing a higher level of adherence [21]. Therefore, added to what can be a positive clinical effect explained by the anti-leukotriene mechanism of montelukast, a greater adherence could be responsible for the effect achieved in the group of older adults, so, in this specific group, its use should be recommended as an integral part of the treatment. This conclusion has also been proposed by the Bozek group, when they stated that the results of their research could be explained, in addition to the anti-leukotriene effect, by a greater adherence to montelukast, compared with the adherence to the inhaled corticosteroid [16].

Regarding the safety findings of montelukast in this cohort of patients, the results corroborate that it is a drug with an adequate safety profile in the adult population, including patients over 60 years of age. These results have been widely corroborated in other studies that have evaluated the effectiveness and safety of montelukast in adults [8, 11, 18].

The present investigation is important in terms of providing evidence that complements previous studies that have demonstrated the clinical effectiveness of montelukast for the treatment of asthma, either as monotherapy or in combination treatment [11]. However, the main contribution is that it is a study conducted in real life conditions and that the effect was compared in elderly patients, for whom a differential effect could be established, which can be attributed to the physiopathogenesis of the disease in this age group, but that could also be associated with greater potential adherence.

Observational studies are susceptible to incur biases that affect the internal validity of their results, constituting their main weakness or limitation [22]. Nevertheless, comparing with controlled clinical trials, they have some advantages, related to the ability to generalize the results, and their external validity [23]. Consequently, it is essential to have a body of evidence from controlled experiments that in a complementary way is complemented with the results of real-life studies that allow corroborating or contrasting the results obtained. It is for this reason that, despite the possible and potential limitations of this study, its results provide fundamental evidence for decision-making in the management of patients with mild or moderate asthma, especially for populations for whom a body of abundant evidence is not available, as are the elderly. Finally, from the present study new opportunities arise for the development of future research, in which patients with other levels of disease severity are included and where it is evaluated whether adherence to therapy modifies the outcomes achieved or simply the effect on the genesis of the disease in the population of asthmatic patients older than 60 years.

## CONCLUSION

In this cohort of patients, treated with montelukast 10 mg/day for 12 weeks, there was a reduction of broncho-obstructive symptoms and exacerbations of the disease. In older adults compared to young adults a greater reduction in the use of beta-2 adrenergic agonist as ***a rescue medication*** and in the concomitant use of inhaled corticosteroid schemes was documented.

## Figures and Tables

**Table 1:** Baseline characteristics in young adults and older adults.

**Finding**	**Young Adults:** ** 18-59 years (n = 74)**		**Older Adults: ** **≥ 60 years (n = 30)**		**Overall**		***p***
	**n**	**%**	**n**	**%**	**n**	**%**	
Last month treatment schedule includes corticosteroids	50	67.5	18	60.0	68	65.4	0.46
Use of rescue inhaled B2 last month	45	61.0	23	77.0	68	65.4	0.12
Attended the emergency room in the last month for asthma	12	16.0	2	7.0	14	13.5	0.19
Wheezing in the consultation	36	48.6	15	50.0	51	49.0	0.9
	**p50**	**IQR**	**p50**	**IQR**	**p50**	**IQR**	***p***
Attack in the last month	2	2	1	1	2	1	0.59
Symptom-free days in the last month	15	6.0	16.5	8.0	15.0	6.5	0.35

**p50**: 50th percentile; **IQR**: Interquartile range (*p*75-p25); ***p***: *p* value.

**Table 2:** Montelukast 10 mg: Baseline evaluation versus 12 weeks of treatment.

**Finding**	**Baseline Evaluation ** **(n = 104)**		**Evaluation 12 Weeks ** **(n = 104)**		***p***
	**n**	**%**	**n**	**%**	
Last month treatment schedule includes corticosteroids	68	65.4	54	51.9	0.048
Use of rescue inhaled B2 last month	68	65.4	51	49.0	0.017
Attended the emergency room in the last month for asthma	14	13.5	9	8.7	0.13
Wheezing in the consultation	51	49.0	30	28.9	0.002
	**p50**	**IQR**	**p50**	**IQR**	**p**
Attacks in the past month	2	1	0	0	0.00
Symptom-free days in the last month	15	6.5	24.5	1.0	0.00

**p50**: 50th percentile; **IQR**: Interquartile range (p75-p25); ***p***: *p* value.

**Table 3:** Montelukast 10 mg-12 weeks post treatment: Young adults Vs. Older adults.

**Finding**	**Young Adults: ** **18-59 Years ** **(n = 74)**		**Older Adults: ** **≥ 60 Years ** **(n = 30)**		**Overall** **(n = 104)**		***p***
	**n**	**%**	**n**	**%**	**n**	**%**	
Last month treatment schedule includes corticosteroids	43	58.1	11	36.7	54	51.9	0.047
Use of rescue inhaled B2 last month	41	55.4	10	33.3	51	49.0	0.041
Attended the emergency room in the last month for asthma	7	9.5	2	6.7	9	8.7	0.64
Wheezing in the consultation	24	32.4	6	20	30	28.9	0.2
	**p50**	**IQR**	**p50**	**IQR**	**p50**	**IQR**	***p***
Attacks in the last month	0	1	0	0	0	0	0.17
Symptom-free days in the last month	24	10.0	27	10.0	24.5	10.0	0.6

**p50**: 50th percentile; **IQR**: Interquartile range (p75-p25); ***p***: *p* value.
